# Efficacy of chlorin dioxide wipes in disinfecting airway devices contaminated with Covid-19

**DOI:** 10.3389/fcimb.2023.1061647

**Published:** 2023-03-22

**Authors:** Hussain Albaharna, Hassan Almubiereek, Mohammed Almualim, Rehab Bukhamsin, Ahmed Abdelfattah, Lamia Buohliqah

**Affiliations:** ^1^ Department of Otolaryngology – Head & Neck Surgery, Qatif Central Hospital, Qatif, Saudi Arabia; ^2^ Intensive Care Department, Dr. Sulaiman Alhabib Hospital/Khobar/Qatif, Khobar, Saudi Arabia; ^3^ Hematopathology Department, Dammam Regional Lab and Blood Bank, Dammam, Saudi Arabia; ^4^ Molecular Department, Dammam Regional Lab and Blood Bank, Dammam, Saudi Arabia

**Keywords:** disinfectant, COVID - 19, airway, endoscopes, chlorine dioxide

## Abstract

**Background:**

Reprocessing and disinfecting endoscopes is a routine practice in otolaryngology. An effective, safe, and rapid disinfection technique is essential during the COVID-19 pandemic

**Objective:**

To validate the efficacy of chlorine dioxide wipes in disinfecting three types of airway endoscopes contaminated with COVID-19-positive patient secretions.

**Methods:**

Chlorine dioxide wipes were tested on rigid nasal endoscopes, laryngoscope blades, and flexible bronchoscopes. The endoscopes were disinfected using the wipes after exposure to COVID-19-positive patients. The tested scope was included in the study if the post procedure pre disinfection swab was positive for COVID-19 virus using RT-PCR. We analyzed 38 samples for 19 subjects (scopes) pre and post disinfection with chlorine dioxide wipes from July 2021 to February 2022.

**Results:**

A total of four rigid endoscopes, four flexible bronchoscopes, and four laryngoscopes were included in the study which represent 24 pre and post disinfection swabs. The others were excluded because of negative pre disinfection swab. All the post disinfection PCR swab results were negative.

**Conclusion:**

Chlorine dioxide is a convenient, fast, and effective disinfection technique for COVID-19-contaminated airway endoscopes.

## Introduction

The authority of the Republic of China had reported pneumonia cases of unknown cause in Wuhan in the province of Hubei (China) between 8 December 2019 and 2 January 2020. However, on 11 and 12 January, they declared the first cases of what is now called SARS-CoV-2, caused by the novel Coronavirus-19 (Johnson, 2020). On 11 February 2020, the WHO announced a name for the new coronavirus disease: COVID-19. By 11 March 2020, the WHO stated that COVID-19 could be characterized as a pandemic (World Health Organization, 2020).

The wide spread of the disease and strict isolation and social distancing protocols strongly affect all aspects of financial and social as well as medical fields (Heckman et al., 2020). Airway instrumentation, especially scoping, is a daily practice in many specialties, such as otolaryngology, anesthesia, intensive medicine, and emergency medicine. Considering the high contiguity of the disease through droplets, this places the physicians in these specialties among those at the highest risk of becoming infected (Krajewska et al., 2020; Vukkadala et al., 2020). The risk is not limited to staff but also applies to patients (Workman et al., 2020). Currently, most otolaryngological societies recommend that diagnostic procedures involving upper airway manipulation, such as anterior rhinoscopy and particularly nasal endoscopy, should be considered high risk for viral transmission and therefore limited to patients with an urgent need for examination during the initial phase of the COVID-19 outbreak (American College of Surgeons, 2020; Davies and Roland, 2020; Van Gerven et al., 2020). This is also applied to other airway procedures performed in other facilities, such as endotracheal intubation and bronchoscopy, particularly those performed by anesthetists and intensivists (Cook et al., 2020; Ti et al., 2020; Wahidi et al., 2020).

Many issues have been raised to address the situation after opening the service. Reprocessing and disinfecting endoscopes are essential after each airway procedure (Van Gerven et al., 2020). Many obstacles may be faced in disinfecting airway endoscopes during the COVID-19 pandemic. The number of endoscopes may be limited, and these must be available for reuse as soon as possible. Transporting COVID-19-contaminated tools can increase the risk of spreading infection between staff. Moreover, transportation may lead to damage or loss of tools, affecting the workflow and productivity as well as incurring costs to repair the damage. An effective, safe, and rapid disinfection technique is essential during the COVID-19 pandemic. The chlorine dioxide wipes is a three-part decontamination system for nonlumened medical devices such as flexible endoscopes, transesophageal echocardio probes (TOE/TEE), transvaginal ultrasound probes, transrectal ultrasound probes, laryngoscope blades, intubation endoscopes, and manometry catheters (Tristel, a). The Wipe is impregnated with (citric acid) Base solution, and the Activator Foam is a dilute sodium chlorite solution. When mixed on applying Activator Foam to the Sporicidal Wipe and scrunching them together, wipe’s proprietary chlorine dioxide chemistry is activated. The Sporicidal Wipe destroys organisms of concern such as bacterial spores, mycobacteria, viruses, fungi, and bacteria within a contact time of 30 seconds (Tristel, b). Thus, it can be applied in different hospital areas, such as outpatient clinics, intensive care units, cardiac laboratories, and radiology departments. Disinfection can be conducted in the same examination area, saving time and effort (Tzanidakis et al., 2012). It involves three wipes, which sequentially perform the decontamination steps within minutes. The first is cleaning with the Pre-Clean Wipe which contains enzymatic detergent, to remove organic matter. The second is high-level disinfection with the Sporicidal Wipe and Activator Foam, and the final step is rinsing with the Rinse Wipe that is impregnated with sterile water to remove any chemical residue from medical device surfaces after high-level disinfection.

This study aimed to validate the efficacy of chlorine dioxide wipes system in disinfecting three types of airway endoscopes contaminated with COVID-19-positive patient secretions.

## Materials and methods

### Study design

This study tested the efficacy of using the chlorine dioxide wipes system. The wipes were tested on three groups of airway endoscopes. The first group was rigid nasal endoscopes after passing them through the nasopharynges of positive patients. Laryngoscope blades in the second group were tested after being used for intubation. Finally, flexible bronchoscopes were tested after bronchoscopic guidance endotracheal intubation. For each group, two swabs were taken from every tested device. The first was immediately after the procedure and the second after disinfection using chlorine dioxide wipes. The swabs were sent for real-time polymerase chain reaction (RT-PCR) tests for COVID-19. A pre disinfection swab was performed to avoid false positive cases. All cleaning episodes were performed by a single person fully trained in the disinfection practice according to the manufacturer’s instructions. After each use, the devices were sent to the central sterile services department CSSD for standard sterilization.

### Protocol for diagnosis of SARS-CoV-2 infection

The currently recommended modality for diagnosing acute or current SARS-CoV-2 infection is a nucleic acid amplification test (NAAT) detecting one or more RNA gene targets specific to the virus. The most common is RT-PCR, which employs fluorescence to detect the amount of amplified DNA in real time (Corman et al., 2020). Some of the most frequently tested gene targets for detecting SARS-CoV-2 include the Envelope E, Spike S, and Nucleocapsid N genes, the RNA-dependent RNA polymerase (RdRp), and open reading frame ORF1a/1b. Detection of the E gene appears to have the highest analytical sensitivity (tested as screening); however, we also tested for another gene target for confirmation. Different NAAT systems vary in terms of clinical sensitivity, regardless of the gene detected. All current SARS-CoV-2 RT-PCR assays with European approval (EUA) are labeled only for qualitative detection of the gene specific to the virus and are not approved for quantitative measurement of the amount of the virus present in the sample. In addition to detecting SARS-CoV-2, many NAATs have been approved as multiplex assays that can simultaneously detect other respiratory viruses, such as influenza a AND b, as well as bacteria causing atypical pneumonia (Chan et al., 2020).

All recommended controls were used: A negative (no template) control was used to exclude sample contamination during the assay run. A positive template (COVID-19_N_P) control was used to verify that the assay run performed as intended and was used on every assay plate.

An internal control was used to detect nucleic acid present in the sample. This also served as an extraction control to ensure that samples tested as negative contained nucleic acid for testing. A negative extraction control (NEC; negative patient sample) served as both a NEC to monitor for any cross-contamination occurring during the extraction process and an extraction control to validate extraction reagents and successful RNA extraction.

This study analyzed 38 samples for 19 subjects (pre and post, for each scope) from July 2021 to February 2022. We used an Applied Biosystems 7500, Real-Time PCR Instrument System and LightCycler 480 (System II; Roche), targeting the genes RdRp, N, RdRp/E, ORF lab, and E.

### Sample size

Guidelines suggested by Julious were followed to estimate the sample size. A minimum sample size of 12 subjects per treatment arm for a main study sample size of 95 participants corresponds to standardized effect sizes of 0.5 (for 90% power based on a standard sample size calculation) (Julious, 2005; Whitehead et al., 2015). The sample was distributed equally across the three groups, with four instruments included from each.

### Inclusion and exclusion criteria

All cases were recruited from hospitalized patients at Qatif Central Hospital. Patients were included if confirmed to be COVID-19-positive by RT-PCR. Additionally, the swab from the instrument used on a patient before disinfection needed to be positive. Conversely, the case was excluded from the study if the swab taken from the instrument after examining the patient and before disinfection was negative for COVID-19.

### Data analysis

Data was entered and analyzed using SPSS (Version 23.0; SPSS Inc., Chicago, IL, USA). The mean and standard deviation were calculated for numerical variables and counts and percentages for categorical variables.

### Ethical considerations

Informed written consent was obtained from each patient. The study was approved by Qatif Central Hospital Scientific Research Ethics Committee (ID: QCH-SRECO 271/2021). Tests were performed with appropriate personal protective equipment to ensure the examiner’s safety. The study protocol was performed in accordance with the relevant guidelines.

## Results

Of all the tested subjects (19 scopes), 7 (14 swabs, 36.8%) were excluded due to negative pre disinfection PCR swab results. A total of 24 swabs (63.2%) from four rigid endoscopes, four flexible bronchoscopes, and four laryngoscopes were included in this study as the pre disinfection PCR swab results were positive. All the post disinfection PCR swab results were negative.

The time consumed during disinfection was between 4 and 5 minutes; the average time required for disinfection was approximately 4:33 minutes. The time between scope infection and starting disinfection ranged from 1 to 2 minutes; we found no difference in the post disinfection results. The disinfection technique between the three different instruments, metal instruments (laryngoscope and rigid nasal scope) and rubber or silicon material fiberoptic nasopharyngoscopy, ran with the same steps, and no post disinfection results were obtained. After each disinfection procedure, the instrument was checked for any damage related to the use of disinfectant including the surface and the lens of the endoscopes. There was no damage documented in all the procedures.

In this study, precise copy-number quantitation of COVID-19 RNA for collected swabs was unnecessary. However, analytical sensitivity and limit of detection (LoD) studies established that the lowest concentration of SARS-CoV-2 (genome copies [cp]/μL) that could be detected by the assays used was Cycle threshold (CT:38). We used CT values of SARS-CoV-2 to detect the sample positivity rather than set clinical cutoffs.

The average CT of all (12) positive swabs ranged from 15 to 34, much lower than 38, which is generally considered an upper borderline for reliable positive results, regardless of the type of commercial RT-PCR test used in SARS-CoV-2 diagnostics. The mean CT value for the positive swabs (pre disinfected) was CT:26 for the RT-PCR results of all tested swabs. All post disinfected swabs showed negative results with no CT detected. (**Table 1**).

**Table 1:** Swab results of each airway tool.

No	Instrument’s type	Pre-disinfection Cycle threshold	pre-result	Post disinfection Cycle threshold	Post-result	Duration of the disinfection (min)
**1**	flexible bronchoscope	None	Negative	None	Negative	4:00
**2**	rigid nasal endoscope	None	Negative	None	Negative	4:30
**3**	flexible bronchoscope	27	Positive	None	Negative	5:00
**4**	rigid nasal endoscope	15	Positive	None	Negative	4:45
**5**	rigid nasal endoscope	30	Positive	None	Negative	4:10
**6**	laryngoscope	None	Negative	None	Negative	4:40
**7**	flexible bronchoscope	29	Positive	None	Negative	4:15
**8**	laryngoscope	20	Positive	None	Negative	4:15
**9**	laryngoscope	None	Negative	None	Negative	4:50
**10**	flexible bronchoscope	34	Positive	None	Negative	4:20
**11**	laryngoscope	None	Negative	None	Negative	4:30
**12**	rigid nasal endoscope	None	Negative	None	Negative	4:50
**13**	flexible bronchoscope	26	Positive	None	Negative	5:00
**14**	rigid nasal endoscope	30	Positive	None	Negative	4:50
**15**	rigid nasal endoscope	None	Negative	None	Negative	4:40
**16**	laryngoscope	28	Positive	None	Negative	4:30
**17**	laryngoscope	25	Positive	None	Negative	4:45
**18**	rigid nasal endoscope	29	Positive	None	Negative	4:20
**19**	laryngoscope	20	Positive	None	Negative	4:30

Note that the tools with pre-disinfection negative results were excluded from the study.

## Discussion

COVID-19 remains a pandemic worldwide. With insufficient vaccination in some parts of the world and the emergence of several new mutations of the virus, conditions are expected to be serious for some time.

This situation raised concerns about sterilization, disinfection, and cleaning in health care environments. These three reprocessing techniques differ in their degree of killing microorganisms. While sterilization eliminates all organisms, disinfection does not eradicate bacterial spores, and cleaning only reduces the burden by removing physical matter. This study tested the efficacy of using chlorine dioxide wipes to disinfect different types of instruments contaminated by secretions from the airways of COVID-19 patients.

Choosing the type of reprocessing technique depends on two factors: the category of hospital instruments required and the manufacturers’ recommendations. Critical items are used on sterile tissue, so sterilization is the ideal reprocessing technique for these instruments. Different airway instruments, such as nasal endoscopes, laryngeal blades, and fiberoptic bronchoscopes, are semi critical items requiring a high level of disinfection (Kothekar and Kulkarni, 2020). Choosing the type of disinfectant depends on several factors, such as the manufacturers’ recommendations, the availability of disinfectant, and the targeted organism. COVID-19 can vary in its persistence on instruments depending on the material. This ranges from around 4 hours on copper to 6 days on plastic materials (Fathizadeh et al., 2020). Many options are available for disinfection against COVID-19. Ultraviolet irradiation, alcohol, hydrogen peroxide, chlorine-containing disinfectants, and others are identified in the literature for use against COVID-19 in different spaces in health care facilities (Sharafi et al., 2021). Among these, chlorine-containing disinfectants are the most widely used; 0.1% sodium hypochlorite was found to be effective in inactivating COVID-19 on contaminated surfaces. It was also used during this pandemic to decontaminate the hands of patients with upper limb injuries (De Vitis et al., 2020). This study has shown that chlorine dioxide wipes system is effective in disinfecting different airway instruments. Moreover, it appears efficient in different contaminated environments as the viral load increases in the throat, nose, and trachea respectively. It is easy and fast to use. This gives it a major advantage in repeated disinfection in the same procedural area. Accordingly, it avoids transporting COVID-19-contaminated instruments, which could transmit infection. Additionally, this increases the life expectancy of the instruments and avoids damage resulting from unnecessary sterilization.

## Conclusion

Chlorine dioxide wipes system is a convenient, fast disinfection technique for COVID-19-contaminated airway endoscopes. This study showed its high efficacy. It is very helpful to be used with many advantages especially in the pandemic situation. Further larger studies should be conducted to support this result.

## Data availability statement

The raw data supporting the conclusions of this article will be made available by the authors, without undue reservation.

## Ethics statement

The studies involving human participants were reviewed and approved by Qatif Central Hospital research and ethical committee. The patients/participants provided their written informed consent to participate in this study.

## Author contributions

All authors listed have made a substantial, direct, and intellectual contribution to the work and approved it for publication.

## References

[B1] American College of Surgeons (2020). COVID 19: Elective case triage guidelines for surgical care. Am. Coll. Surg. 24, 11–13.

[B2] Chan J. F.-W. Yip C. C.-Y. To K. K.-W. Tang T. H.-C. Wong S. C.-Y. Leung K.-H.. (2020). Improved molecular diagnosis of COVID-19 by the novel, highly sensitive and specific COVID-19-RdRp/Hel real-time reverse transcription-PCR assay validated *In vitro* and with clinical specimens. J. Clin. Microbiol. 58 (5), e003310–20. doi: 10.1128/JCM.00310-20 PMC7180250 32132196

[B3] Cook T. M. El-Boghdadly K. McGuire B. McNarry A. F. Patel A. Higgs A. (2020). Consensus guidelines for managing the airway in patients with COVID-19: Guidelines from the difficult airway society, the association of anaesthetists the intensive care society, the faculty of intensive care medicine and the royal college of anaesthetist. Anaesthesia 75 (6), 785–799. doi: 10.1111/anae.15054 32221970 PMC7383579

[B4] Corman V. M. Landt O. Kaiser M. Kaiser M. Molenkamp R. Meijer A.. (2020). Detection of 2019 novel coronavirus (2019-nCoV) by real-time RT-PCR. Euro Surveill. 25 (3), 23–30. doi: 10.2807/1560-7917.ES.2020.25.3.2000045 PMC6988269 31992387

[B5] Davies E. Roland N. (2020) Nasal endoscopy and laryngoscopy examination of ENT patients. Available at: http://orlcg.me/files/documents/b4967457-3475-4c2f-b1a5-621a5fb0b1be.pdf (Accessed 2022 July 16).

[B6] De Vitis R. Passiatore M. Perna A. Proietti L. Taccardo G. (2020). COVID-19 contagion and contamination through hands of trauma patients: What risks and what precautions? J. Hosp. Infect. 105 (2), 354–355. doi: 10.1016/j.jhin.2020.03.037 32259547 PMC7129819

[B7] Fathizadeh H. Maroufi P. Momen-Heravi M. Dao S. Köse Ş Ganbarov K.. (2020). Protection and disinfection policies against SARS-CoV-2 (COVID-19). Le Infez Med. 28 (2), 185–191. 32275260

[B8] Heckman G. A. Saari M. McArthur C. Wellens N. I. H. Hirdes J. P. (2020). COVID-19 outbreak measures may indirectly lead to greater burden on hospitals. Can. Med. Assoc. J. 192 (14), E384–E384. doi: 10.1503/cmaj.75230 32392532 PMC7145375

[B9] Johnson M. (2020). Wuhan 2019 novel coronavirus - 2019-nCoV. Mater. Methods 10, 1–5. doi: 10.13070/mm.en.10.2867

[B10] Julious S. A. (2005). Sample size of 12 per group rule of thumb for a pilot study. Pharm. Stat. 4 (4), 287–291. doi: 10.1002/pst.185

[B11] Kothekar A. T. Kulkarni A. P. (2020). Basic principles of disinfection and sterilization in intensive care and anesthesia and their applications during COVID-19 pandemic. Indian J. Crit. Care Med. 24 (11), 1114–1124. doi: 10.5005/jp-journals-10071-23562 33384520 PMC7751027

[B12] Krajewska J. Krajewski W. Zub K. Zatoński T. (2020). COVID-19 in otolaryngologist practice: A review of current knowledge. Eur. Arch. Otorhinolaryngol. 277 (7), 1885–1897. doi: 10.1007/s00405-020-05968-y 32306118 PMC7166003

[B13] Sharafi S. M. Ebrahimpour K. Nafez A. (2021). Environmental disinfection against COVID-19 in different areas of health care facilities: A review. Rev. Environ. Health 36 (2), 193–198. doi: 10.1515/reveh-2020-0075 32845869

[B14] Ti L. K. Ang L. S. Foong T. W. Ng B. S. W. (2020). What we do when a COVID-19 patient needs an operation: Operating room preparation and guidance. Can. J. Anesth. 67 (6), 756–758. doi: 10.1007/s12630-020-01617-4 32144591 PMC7090746

[B15] Tristel Tristel duo OPH. (2020). Available at: https://www.tristel.com/uk/tristel-products/tristel-duo-oph (Accessed 2022 September 16).

[B16] Tristel Trio wipes system. (2020). Available at: https://tristel.com/au-en/wp-content/uploads/sites/21/2020/12/Mkt-Bro-1331-1-Tristel-Trio-Wipes-System-Brochure-Laryngoscope-Blades-EN.pdf (Accessed 2022 September 16).

[B17] Tzanidakis K. Choudhury N. Bhat S. Weerasinghe A. Marais J. (2012). Evaluation of disinfection of flexible nasendoscopes using tristel wipes: A prospective single blind study. Ann. R Coll. Surg. Engl. 94 (3), 185–188. doi: 10.1308/003588412X13171221589937 22507724 PMC3705233

[B18] Van Gerven L. Hellings P. W. Cox T. Fokkens W. J. Hopkins C. Fokkebs W. J.. (2020). Personal protection and delivery of rhinologic and endoscopic skull base procedures during the COVID-19 outbreak. Rhinol. J. 58 (3), 289–294. doi: 10.4193/rhin20.119 32441710

[B19] Vukkadala N. Qian Z. J. Holsinger F. C. Patel Z. M. Rosenthal E. (2020). COVID-19 and the otolaryngologist - preliminary evidence-based review. Laryngoscope 130 (11), 2537–2543. doi: 10.1002/lary.28672 32219846

[B20] Wahidi M. M. Lamb C. Murgu S. Musani A. Shojaee S. Sachdeva A.. (2020). American Association for bronchology and interventional pulmonology (AABIP) statement on the use of bronchoscopy and respiratory specimen collection in patients with suspected or confirmed COVID-19 infection. J. Bronchol. Interv. Pulmonol. 27 (4), e52–e54. doi: 10.1097/LBR.0000000000000681 PMC7141581 32195687

[B21] Whitehead A. L. Julious S. A. Cooper C. L. Campbell M. J. (2015). Estimating the sample size for a pilot randomised trial to minimise the overall trial sample size for the external pilot and main trial for a continuous outcome variable. Stat. Methods Med. Res. 25 (3), 1057–1073. doi: 10.1177/0962280215588241 26092476 PMC4876429

[B22] Workman A. D. Welling D. B. Carter B. S. Curry W. T. Holbrook E. H. Gray S. T.. (2020). Endonasal instrumentation and aerosolization risk in the era of COVID-19: Simulation, literature review, and proposed mitigation strategies. Int. Forum Allergy Rhinol. 10 (7), 798–805. doi: 10.1002/alr.22577 32243678

[B23] World Health Organization (2020) Archived: WHO timeline - COVID-19. Available at: https://www.who.int/news/item/27-04-2020-who-timeline—covid-19 (Accessed 2022 July 16).

